# Endoscopic retrograde cholangiopancreatography in patients with surgically altered anatomy: One single center's experience

**DOI:** 10.1097/MD.0000000000005743

**Published:** 2016-12-30

**Authors:** Fei Wang, Boming Xu, Quanpeng Li, Xiuhua Zhang, Guobing Jiang, Xianxiu Ge, Junjie Nie, Xiuyun Zhang, Ping Wu, Jie Ji, Lin Miao

**Affiliations:** a Medical Center for Digestive Diseases, The Second Affiliated Hospital of Nanjing Medical University; b Liver Transplantation Center, The First Affiliated Hospital of Nanjing Medical University, Nanjing, Jiangsu Province, China.

**Keywords:** altered gastrointestinal anatomy, Billroth, double-balloon enteroscope, endoscopic retrograde cholangiopancreatography, Roux-en-Y anastomosis

## Abstract

Endoscopic retrograde cholangiopancreatography (ERCP) in patients with surgically altered anatomy is challenging. Results of ERCP in those patients varied.

The aim of our study was to evaluate the safety and effectiveness of various endoscopes-assisted ERCP in patients with surgically altered anatomy.

Fifty-two patients with Billroth II reconstruction (group A), 20 patients with subtotal or total gastrectomy with Roux-en-Y anastomosis (group B), 25 patients with pancreatoduodenectomy or Roux-en-Y hepaticojejunostomy reconstruction (group C) were included. Gastroscope, duodenoscope, colonoscope, and double-balloon enteroscope were used.

The endoscope insertion success rate of groups A, B, C was 96.2% (50/52), 85.0% (17/20), 80% (20/25), respectively. *χ*^*2*^ test showed that there was no significant difference between the 3 groups (*P* = 0.068). The mean insertion time was 36.7, 68.4, and 84.0 minutes, respectively. One-way ANOVA showed that the insertion time of group C was significantly longer than that of groups B and C (both *P* <0.001). The endoscopic cannulation success rates of groups A, B, C were 90%, 82.4%, and 100%, respectively. *χ*^*2*^ test showed that there was no significant difference between the 3 groups (*P* = 0.144). The mean cannulation time was 19.4, 28.1, and 20.4 minutes, respectively. One-way ANOVA showed that the cannulation time of group B was longer than that of groups A and C (*P* <0.001, *P* = 0.001, respectively). In total, 74 patients with successful biliary cannulation achieved the therapeutic goal; thus, the clinical success rate was 76.3% (74/97).

Our study showed that ERCP in patients with surgically altered anatomy was safe and feasible.

## 1 Introduction

Since endoscopic retrograde cholangiopancreatography (ERCP) was first reported in 1968, it has been widely used for the diagnosis and therapy of pancreatobiliary diseases.^[1,2]^ In recent years, with the continuous development of endoscopy and interventional radiology techniques, ERCP has developed rapidly in pancreatobiliary diseases. The success rate of ERCP in patients with normal gastrointestinal anatomy has been estimated to be 95%.^[3]^ However, for patients with surgically altered anatomy, ERCP becomes difficult and challenging, particularly in patients who have undergone Billroth II reconstruction, subtotal or total gastrectomy with Roux-en-Y anastomosis, pancreatoduodenectomy or Roux-en-Y hepaticojejunostomy reconstruction and Roux-en-Y gastric bypass. The ERCP procedure for patients with surgically altered anatomy includes access to the afferent limb, reaching the papilla or bilioenteric/pancreatoenteric anastomosis, and cannulation of pancreatobiliary system and endoscopic treatment.^[4,5]^

Since double-balloon enteroscope (DBE) for the diagnosis and treatment of small intestinal lesions was described in 2001,^[6]^ and DBE-assisted ERCP was first successfully performed in a patient with Roux-en-Y choledochojejunostomy reconstruction in 2005,^[7]^ great progress has been made for ERCP in patients with altered gastrointestinal anatomy, but the results varied.^[8]^ Especially, limited data are available on the outcome of balloon enteroscopy-assisted ERCP in patients with surgically altered anatomy.

In the present study, we evaluated the safety and effectiveness of various endoscopes-assisted ERCP in patients with surgically altered anatomy.

## 2 Materials and methods

### 2.1 Patients

A total of 97 patients with surgically altered anatomy were referred to our center between January 2013 and January 2016. All the patients were candidates for endoscopic therapy based on computed tomography, magnetic resonance cholangiopancreatography, and endoscopic ultrasonography findings.

The surgically altered anatomy included Billroth II reconstruction, subtotal or total gastrectomy with Roux-en-Y anastomosis, pancreatoduodenectomy or Roux-en-Y hepaticojejunostomy reconstruction.

The patient database included data pertaining to patient demographics, the details of endoscope insertion, endoscopic cannulation and endoscopic treatment, complications, and so on.

This study was approved by the Medical Center for Digestive Diseases, Second Affiliated Hospital of Nanjing Medical University at Nanjing, China. Institutional review board approval was obtained for this retrospective study. Written informed consent was obtained from each patient before ERCP.

### 2.2 Methods

All ERCPs were performed with patients under conscious sedation by using intravenous remifentanil and dexmedetomidine. ERCP was started with the patient in the prone position or in the left lateral position. All patients were routinely supplied with oxygen (2 L/min) via a nasal prong. Patient vital signs, including heart and respiration rates, electrocardiography, and pulse oximetry, were monitored continuously during the ERCP procedure. The procedures were performed by 3 experienced endoscopists (LM, QL, and XZ) who have performed more than 500 ERCPs.

A commercially available duodenoscope (TJF 260 V, Olympus, Tokyo, Japan), gastroscope (GIF Q260J/Q260/H260, Olympus), standard colonoscope (CF HQ260/H260AI, Olympus), long-type colonoscope (CF H260AL, Olympus), and double-balloon enteroscope (EN-450T5, Fujifilm, Tokyo, Japan) was used in this study. A transparent cap (D-201-11802, Olympus) was attached to the tip of the gastroscope and colonoscope to improve the visualization of endoscope insertion and to facilitate endoscopic cannulation.

When the intact papilla, bilioenteric/pancreatoenteric anastomosis was reached, selective biliary cannulation was tried with a catheter (StarTipV, PR-V434Q, Olympus). Selective cannulation was performed using the wire-guided cannulation method with a 0.035-inch guidewire (Jagwire, Boston Scientific, Natick, MA). When a long-type colonoscope or double-balloon enteroscope was used, a prototype catheter (Prototype, JIUHONG Medical Instrument Co, Ltd, Changzhou, China) was used for endoscopic cannulation. Endoscopic papillary balloon dilation was performed using a balloon dilator (CRE Balloon Dilator; Boston Scientific).

When selective biliary cannulation failed and the pancreatic duct was cannulated, the double-guidewire technique (DGT) was used with another guidewire. DGT was performed as follows: a guidewire was inserted into the pancreatic duct, then a sphincterotome (TRI-25M-P, Wilson-Cook, NC, USA) was reinserted along the first guidewire after being reloaded with the second guidewire to attempt cannulation of the bile duct. After successful biliary cannulation, the pancreatic wire was removed. Otherwise, the precut technique was performed by using a sphincterotome or a needle-knife (KD-441Q or KD-10Q-1, Olympus).

**Figure 1:**
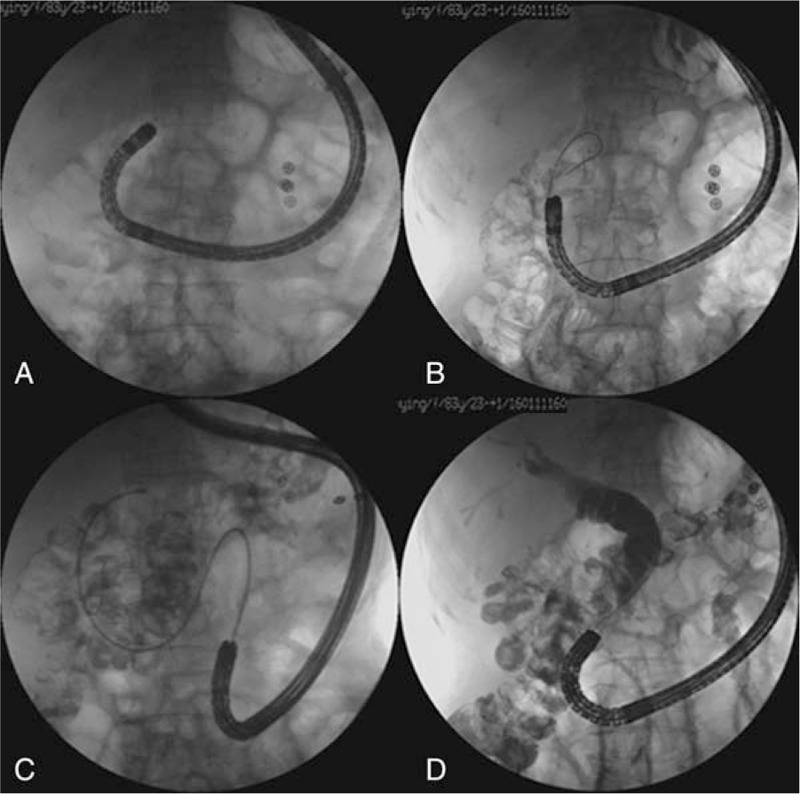
The procedure of endoscope exchange technique. A, Advancing a forward-viewing endoscope to the papilla, but the cannulation failed. B, Placing a guidewire to the biliopancreatic limb, then the forward-viewing endoscope was withdrawn leaving the guidewire in place. C, A duodenoscope was advanced over the guidewire to reach the papilla. D, Selective cannulation was achieved successfully.

When the cannulation with forward-viewing endoscope failed after precut or DGT, we used 1 special method called endoscope exchange technique. The technique involves advancing a forward-viewing endoscope to the papilla, followed by placing a guidewire in the biliopancreatic limb, then a duodenoscope can be advanced over the wire and cannulating into the bile duct. The procedure of endoscope exchange technique is shown in Fig. 1.

When biliary cannulation could not be achieved despite the use of various techniques for approximately 40 minutes, the procedure was terminated. We then performed the percutaneous transhepatic cholangiography (PTC)-guided rendezvous technique, followed by the transpapillary approach. In case of biliary stricture, a plastic stent (Flextent, Garson, Changzhou, China) or self-expandable metal stent (Wallstent, Boston Scientific) was inserted.

### 2.3 Definitions

Endoscope insertion success was defined as successful reaching the papilla or bilioenteric/pancreatoenteric anastomosis. Endoscopic cannulation success was defined as deep cannulation into the target duct. ERCP success was defined as successful access to the pancreatobiliary system and its selective cannulation. Clinical success was defined as the selective cannulation and achievement of the planned therapeutic goal.

Cannulation in which advanced methods (such as precut, DGT, endoscope exchange technique, and PTC-guided rendezous technique) were not used was regarded as standard cannulation.

The procedure time of endoscope insertion was measured from the endoscope introducing through the mouth to reaching the pancreatobiliary system. The procedure time of endoscopic cannulation was measured from the first attempt to the end of cannulation.

ERCP-related adverse events included pancreatitis, hyperamylasemia, perforation, bleeding, cholangitis, and cardiopulmonary adverse events.^[9,10]^ Post-ERCP pancreatitis was defined as persistent abdominal pain with the serum amylase value exceeded 3 times the upper limit of normal within 24 hours after ERCP. Hyperamylasemia was defined as the elevation of serum amylase level without typical abdominal pain. Bleeding was defined as clinical evidence of bleeding that required intervention or blood transfusion. Perforation was defined as subcutaneous emphysema, retroperitoneal air, or subphrenic free which was detected during or after ERCP. Cholangitis was defined as right upper quadrant pain accompanied by fever >38.5°C, as well as white blood cell count >10 × 10^9^/L without infectious lesions within 24 hours after ERCP. Cardiopulmonary adverse events included hypoxia, blood pressure drop, shock, myocardial ischemia, or myocardial infarction.

### 2.4 Statistical analysis

The *χ*^*2*^ test or the Fisher exact test was used to compare categorical variables, and the Student *t* test was used to compare continuous variables. Comparisons were carried out using the 1-way ANOVA. A *P* <0.05 was considered to be statistically significant. Statistical analysis was performed using statistical software (SPSS version 17.0, SPSS, Chicago, IL).

## 3 Results

### 3.1 Baseline characteristics

A total of 97 patients with surgically altered anatomy underwent ERCP in our center from January 2013 to January 2016. Among them, 52 patients (mean age 68.1 years, range 41–84 years; 41 males, 11 females) had Billroth II reconstruction, 20 patients (mean age 60.7 years, range 36–83 years; 8 males, 12 females) had subtotal or total gastrectomy with Roux-en-Y anastomosis, 25 patients (mean age 55.3 years, range 28–79 years; 10 males, 15 females) had pancreatoduodenectomy or Roux-en-Y hepaticojejunostomy reconstruction. Demographic characteristics are shown in Table 1.

**Table 1:**
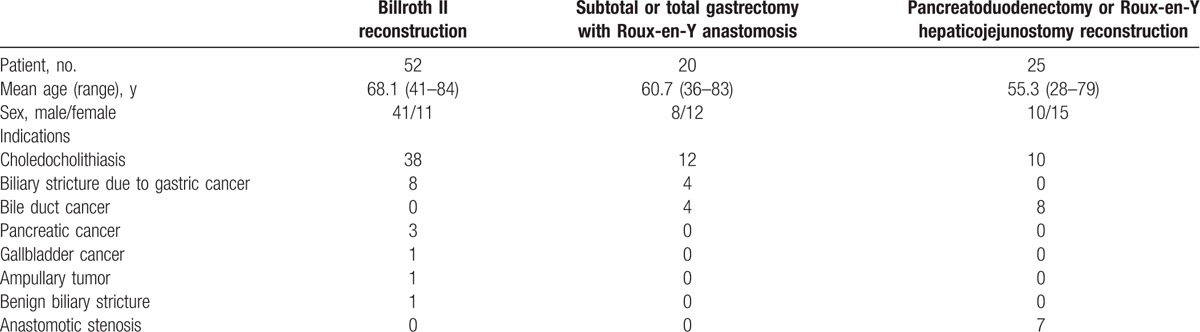
Patient characteristics.

### 3.2 Results of endoscope insertion

In our study, we used gastroscope, duodenoscope, standard colonoscope, long-type colonoscope, and double-balloon enteroscope to perform ERCP, according to patients’ postoperative anatomy and the endoscopist's experience. The results of endoscope insertion are shown in Table 2.

**Table 2:**
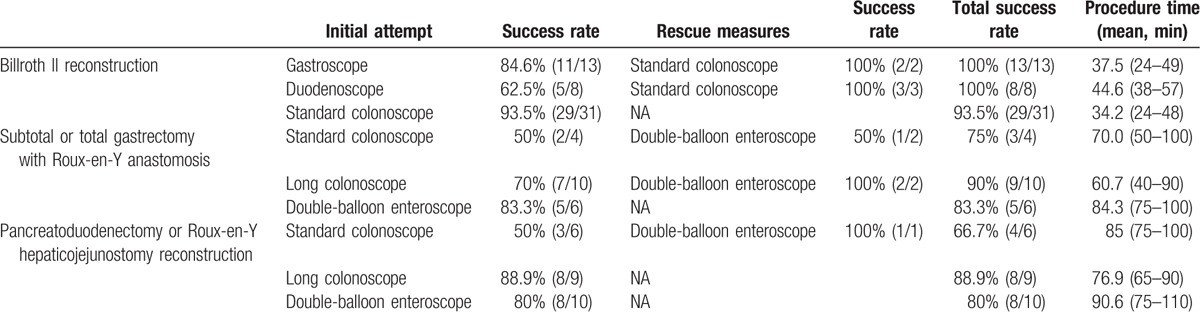
Outcomes of endoscope insertion.

For the 52 patients with Billroth II reconstruction, gastroscope was used in 13 patients, the successful rate of initial endoscope insertion was 84.6% (11/13). For the 2 patients in whom the insertion of a gastroscope was unsuccessful, a standard colonoscope was used at the second attempt, and the papilla was reached successfully. Duodenoscope was used in 8 patients, the successful rate of initial endoscope insertion was 62.5% (5/8). For the 3 patients in whom the insertion of a duodenoscope was unsuccessful, a standard colonoscope was used at the second attempt, and the papilla was reached successfully. Standard colonoscope was used in 31 patients, the successful rate of initial endoscope insertion was 93.5% (29/31). For the 2 patients in whom the insertion of a standard colonoscope was unsuccessful, 1 with common bile duct stone underwent surgery, the other one underwent conservative therapy because of tumor recurrence. By *χ*^*2*^ test, there was no significant difference in the insertion success rates between the 3 types of endoscope (*P* = 0.07).

The mean procedure time of endoscope insertion was 37.5, 44.6, 34.2 minutes for gastroscope, duodenoscope, standard colonoscope, respectively. By 1-way ANOVA, the procedure time of gastroscope and standard colonoscope was shorter than that of duodenoscope (*P* = 0.041, 0.001, respectively). While there was no significant difference in the procedure time between gastroscope and standard colonoscope (*P* = 0.199).

For the 20 patients with subtotal or total gastrectomy with Roux-en-Y anastomosis, the initial endoscope insertion success rate of standard colonoscope, long-type colonoscope, and double-balloon enteroscope was 50% (2/4), 70% (7/10), 83.3% (5/6), respectively. For the 2 patients in whom the insertion of a standard colonoscope and 2 patients in whom the insertion of a long-type colonoscope were unsuccessful, a double-balloon enteroscope was used at the second attempt, and the papilla was reached successfully in 1 (1/2, 50%) patient and 2 (2/2, 100%) patients, respectively. *χ*^*2*^ test showed no significant difference in the initial insertion success rate between them (*P* = 0.684).

The mean insertion time of standard colonoscope, long-type colonoscope, and double-balloon enteroscope was 70.0, 60.7, 84.3 minutes, respectively. One-way ANOVA showed that there was no significant difference in the insertion time between them (*P* = 0.114).

For the 25 patients with pancreatoduodenectomy or Roux-en-Y hepaticojejunostomy reconstruction, the initial endoscope insertion success rate of standard colonoscope, long-type colonoscope, and double-balloon enteroscope was 50% (3/6), 88.9% (8/9), 80% (8/10), respectively. For the 1 patient in whom the insertion of a standard colonoscope was unsuccessful, a double-balloon enteroscope was used at the second attempt, and the papilla was reached successfully. *χ*^*2*^ test showed no significant difference in the initial insertion success rate between them (*P* = 0.305).

The mean insertion time of standard colonoscope, long-type colonoscope, and double-balloon enteroscope was 85.0, 76.9, 90.6 minutes, respectively. By 1-way ANOVA, the procedure time of long-type colonoscope was shorter than that of double-balloon enteroscope (*P* = 0.014). While there was no significant difference in the procedure time between standard colonoscope and long-type colonoscope (*P* = 0.199).

We further analyzed the overall endoscope insertion success rate and the insertion time of patients with different postoperative anatomy. The overall endoscope insertion success rate of patients with Billroth II reconstruction, subtotal or total gastrectomy with Roux-en-Y anastomosis and pancreatoduodenectomy or Roux-en-Y hepaticojejunostomy reconstruction was 96.2% (50/52), 85.0% (17/20), 80% (20/25), respectively, and the mean insertion time was 36.7, 68.4, and 84.0 minutes, respectively. *χ*^*2*^ test showed that there was no significant difference in the overall endoscope insertion success rate between patients with different postoperative anatomy (*P* = 0.068). The 1-way ANOVA showed that the insertion time of patients with Billroth II reconstruction was shorter than that of patients with subtotal or total gastrectomy with Roux-en-Y anastomosis and pancreatoduodenectomy or Roux-en-Y hepaticojejunostomy reconstruction (both *P* <0.001). Moreover, the insertion time of patients with pancreatoduodenectomy or Roux-en-Y hepaticojejunostomy reconstruction was significantly longer than that of patients with subtotal or total gastrectomy with Roux-en-Y anastomosis (*P* <0.001).

There were 10 patients with unsuccessful endoscope insertion in our study. The main reasons included adhesions, sharp angulations, extremely long limbs, and failing to find the bilioenteric/pancreatoenteric anastomosis. Among them, 5 underwent surgery, 3 underwent percutaneous transhepatic biliary drainage, and 2 were treated conservatively.

### 3.3 Results of endoscopic cannulation

The results of endoscopic cannulation are shown in Table 3.

**Table 3:**
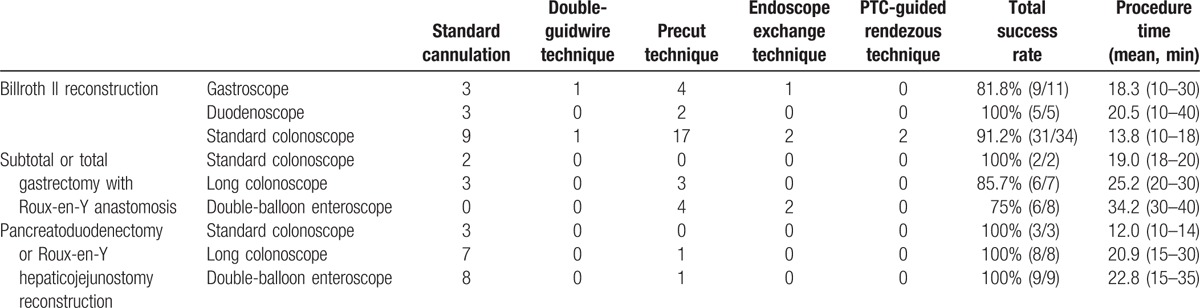
Results of endoscopic cannulation.

Among the 50 patients with Billroth II reconstruction and successful papilla access, selective biliary cannulation was achieved in 45 patients (90%), giving an overall ERCP success rate of 86.5% (45/52). Of the 45 patients, 15 underwent the standard cannulation technique, 23 underwent the precut technique, 2 underwent the double-guidwire technique, and 2 underwent the PTC-guided rendezvous technique. Of the other 3 cases, 1 with gastroscope insertion and 2 with standard colonoscope insertion, cannulation was unsuccessful initially by using standard cannulation technique and precut technique, then the endoscope exchange technique was used with a duodenoscope, achieving successful cannulation finally.

There were 17 patients with subtotal or total gastrectomy with Roux-en-Y anastomosis in whom the papilla was reached. Selective biliary cannulation was achieved in 82.4% (14/17). The ERCP success rate was 70.0% (14/20). Among them, the standard cannulation technique was performed in 5 patients, the precut technique was performed in 7 patients. Two patients with double-balloon enteroscope insertion failed to selective biliary cannulation even by using standard cannulation technique and precut technique, then the endoscope exchange technique was performed, both of them achieved successful cannulation by using standard colonoscope instead.

For the 25 patients with pancreatoduodenectomy or Roux-en-Y hepaticojejunostomy reconstruction, 20 reached the papilla and selective biliary cannulation was successful in 100% (20/20). Thus, the ERCP success rate was 80.0% (20/25). Of the 20 patients, 18 underwent the standard cannulation technique, 2 underwent the precut technique.

The mean cannulation time of patients with Billroth II reconstruction, subtotal or total gastrectomy with Roux-en-Y anastomosis and pancreatoduodenectomy or Roux-en-Y hepaticojejunostomy reconstruction was 19.4, 28.1, and 20.4 minutes, respectively. The 1-way ANOVA showed that the cannulation time of patients with subtotal or total gastrectomy with Roux-en-Y anastomosis was longer than that of patients with billroth II reconstruction and pancreatoduodenectomy or Roux-en-Y hepaticojejunostomy reconstruction (*P* <0.001, *P* = 0.001, respectively). But there was no significant difference in the cannulation time between patients with billroth II reconstruction and pancreatoduodenectomy or Roux-en-Y hepaticojejunostomy reconstruction (*P* = 0.558).

In our study, a total of 8 patients failed to achieve selective cannulation, because of poor en face visualization of the papilla (5/8, 62.5%) and the presence of a peripapillary diverticulum (3/8, 37.5%). Of the 8 patients with unsuccessful selective cannulation, 3 underwent percutaneous transhepatic biliary drainage, 3 underwent surgery, and 2 were treated conservatively because of tumor recurrence.

In our study, 74 (74/79, 93.7%) patients with successful biliary cannulation achieved the therapeutic goal, thus the clinical success rate was 76.3% (74/97). The therapeutic interventions included endoscopic sphincterotomy (5/74, 6.8%), stone extraction (25/74, 33.8%), biliary plastic stent placement (13/74, 17.6%), endoscopic metal stent placement (2/74, 2.7%), endoscopic nasobiliary drainage (18/74, 24.3%), endoscopic papillary balloon dilation (11/74, 14.9%). Five patients with successful biliary cannulation failed to perform endoscopic treatment because of the lack of ERCP accessories.

### 3.4 Results of adverse events

There were 10 cases of adverse events, accounting for 10.3% (10/97) of the total cases. The adverse events included 3 pancreatitis (mild to moderate), 4 hyperamylasemia, 1 cholangitis, 1 bleeding, and 1 cardiopulmonary accident. Bleeding happened after the PTC-guided rendezvous technique. The patient with cardiopulmonary accident presented with hypoxia and blood pressure drop during the ERCP procedure. All the patients were managed with conventional therapy. The results of adverse events are shown in Table 4.

**Table 4:**
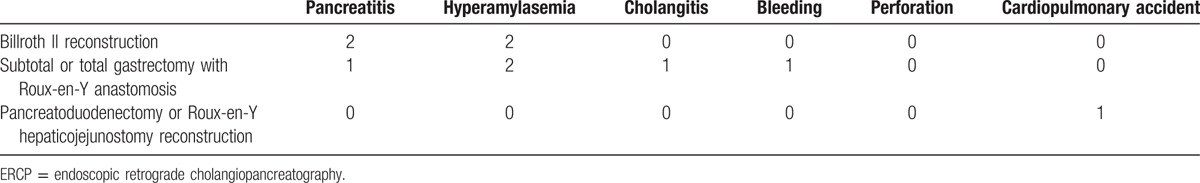
Outcomes of ERCP complications.

## 4 Discussion and conclusions

ERCP in patients with surgically altered anatomy is challenging, sometimes even impossible with a conventional side-viewing duodenoscope. For those special patients, ERCP faces 3 important challenges determining the procedure's success rate: reaching the papilla or the bilioenteric/pancreatoenteric anastomosis, cannulation of the biliopancreatic system, and performing therapeutic interventions, control of possible complications.^[11,12]^

Until now, there are no standardized practical guidelines on this topic. Before performing an ERCP in the patient with surgically altered anatomy, the endoscopist should have a clear understanding of the common surgical rearrangements and the patient's specific surgical history. The success rates, risks, benefits, and planned sequence of alternatives to performance of ERCP in altered anatomy should be thoroughly reviewed with the patient and family.

**Figure 2:**
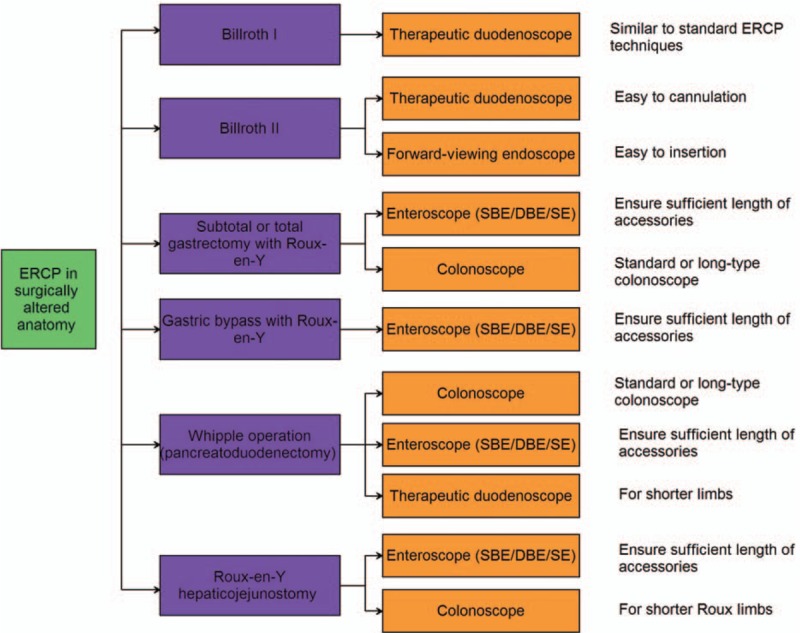
The flow chart of endoscope selection for ERCP in surgically altered anatomy. ERCP = endoscopic retrograde cholangiopancreatography.

Duodenoscope, gastroscope, colonoscope, and enteroscope have been used in different patients with surgically altered anatomy. Endoscope selection is largely based on the patient's postoperative anatomy, including the lengths of afferent, efferent, or Roux limbs, and the type of biliary drainage present (intact papilla or bilioenteric/pancreaticoenteric anastomosis), accessories availability and the endoscopist’ experience.^[8]^ The recommendations of endoscope selection are shown in Fig. 2.

A conventional side-viewing duodenoscope can be used in a case of short-limb postoperative anatomy.^[13]^ However, due to its limited visibility (side view, not forward view), rigidity, and relatively large diameter, crossing the anastomosis and angulation is difficult and increasing the risk of perforation at the level of the anastomosis or the afferent limb.^[14]^ Forward-viewing gastroscopes and colonoscopes, with or without additional distal cap, have been shown to be useful from previous studies.^[15]^

In our study, we used a distal cap at the tip of the gastroscope and colonoscope to improve the visualization of endoscope insertion and to help cannulation since it enables tilting of the papilla.^[16,17]^

For patients with Roux-en-Y anastomosis, longer endoscopes like device-assisted enteroscopy (single-balloon, double-balloon, and spiral enteroscopy) are usually necessary to intubate the afferent limb because of the lengthy limbs.^[18,19]^ In our study, we used double-balloon enteroscope for patients with long limbs.

Additionally, many mew equipments have been used in patients with surgically altered anatomy. Prototype endoscopes like the swan neck shaped multibending backward-oblique viewing duodenoscope (M-D scope, TJF-Y0011; Olympus),^[20]^ the variable stiffness duodenoscope (TJF-Y0001; Olympus),^[21]^ and the multibending forward-viewing endoscope with 2 working channels (M-scope, GIF-2T260 M, Olympus)^[22]^ have been reported and may increase the ERCP success rate in the future.

During the ERCP procedure, identification of the afferent limb may be the first challenge. Usually, the afferent limb can be recognized based on the presence of luminal bile and antiperistaltic motility. Besides, fluoroscopy during the endoscope insertion is helpful to identify the afferent limb by heading toward the upper abdomen. Air enterogram by insufflation of a closed loop system is another method to estimate the direction of the afferent limb and the distance toward the biliopancreatic system.^[19]^ Yano et al^[23]^ used an intraluminal injection of indigocarmine to identify the afferent limb, a method that had a success rate of 80%. This method may be useful in the identification of the afferent limb.^[23]^

In recent years, the success rate of reaching the papilla or biliopancreatoenteric anastomosis in patients with surgically altered anatomy, particularly those after Roux-en-Y reconstruction including gastric bypass and total or subtotal gastrectomy, has increased significantly owing to the development of double-balloon enteroscopy and single-balloon enteroscopy.^[24]^ Until now, there appears to be no difference in the success rate of entering the afferent limb between all 3 device-assisted enteroscopy methods (single-balloon, double-balloon, and spiral enteroscopy).^[25–27]^

In the present study, we used gastroscope, duodenoscope, and standard colonoscope for patients with billroth II reconstruction, standard colonoscope, long-type colonoscope, and double-balloon enteroscope for patients with subtotal or total gastrectomy with Roux-en-Y anastomosis and pancreatoduodenectomy or Roux-en-Y hepaticojejunostomy reconstruction. Our results showed that there was no significant difference in the endoscope insertion success rate between different endoscopes. What is more, the endoscope insertion success rate of patients with Billroth II reconstruction, subtotal, or total gastrectomy with Roux-en-Y anastomosis and pancreatoduodenectomy or Roux-en-Y hepaticojejunostomy reconstruction was 96.2%, 85.0%, 80%, respectively. There was no significant difference in the endoscope insertion success rate between patients with different postoperative anatomy (*P* = 0.068).

Our results also showed that the insertion time of patients with pancreatoduodenectomy or Roux-en-Y hepaticojejunostomy reconstruction was significantly longer than that of patients with subtotal or total gastrectomy with Roux-en-Y anastomosis (*P* <0.001). It may be associated with the long time spend in finding the bilioenteric/pancreatoenteric anastomosis during the endoscope insertion. Unfortunately, details of finding the bilioenteric/pancreatoenteric anastomosis were not systematically collected.

When the papilla was reached, cannulation of a native papilla is particularly difficult because of the oblique and inverted endoscopic view of the papilla, limited availability of accessories, and lack of an elevator.^[24]^

In our experience, obtaining a favorable view of the papilla is considered to be the major factor for difficult cannulation. That was the reason in 5 patients with unsuccessful biliary cannulation in our study. The distal approach changes the direction of cannulation of papilla because the common bile duct is in direct line with the working channel of the forward-viewing endoscope, in contrast to conventional ERCP in normal anatomy using a side-viewing duodenoscope.^[28]^

A major problem is the limitation of devices and accessories, particularly when a long-type balloon enteroscope is used. In our study, with the use of prototype long devices, the success rate of biliary cannulation by using a double-balloon enteroscope for patients with Roux-en-Y reconstruction and intact papilla was 66.7% (6/9).

For surgically altered anatomy without intact papilla, cannulation of a bilioenteric/pancreatoenteric anastomosis is easier than that of an intact papilla because of the lack of a sphincter in a papillary structure. In our study, the success rate of biliary cannulation for patients with bilioenteric/pancreatoenteric anastomosis was 100% (20/20).

However, identifying the bilioenteric/pancreatoenteric anastomosis may be difficult sometimes. Usually, its location can be found based on the intermittent bile flow in the afferent limb. However, when stenosis occurs at the level of the bilioenteric/pancreatoenteric anastomosis, its location is difficult to find. In this case, fluoroscopy can show the position of the endoscope's tip near the liver or the pancreas. Air cholangiogram with insufflation of the closed afferent limb may help to find the bilioenteric/pancreatoenteric anastomosis. Otherwise, mucosal scar tissue with star shaped folds may direct to the location of the strictured anastomosis.

During the endoscopic cannulation, when the cannulation with forward-viewing endoscope failed after precut or double-guidwire technique, we used 1 special method called endoscope exchange technique. The technique involves advancing a forward-viewing endoscope to the papilla, followed by placing a guidewire in the biliopancreatic limb. Then a duodenoscope can be advanced over the wire to reach the papilla. One previous study showed that the successful insertion rate of duodenoscopes with endoscope exchange technique was 67% (10/15) for patients with long-limb Roux-en-Y anatomy, and the subsequent ERCP success rate was 100% (10/10).^[29]^

In our study, there were 3 patients with billroth II reconstruction in whom the cannulation of a forward-viewing endoscope was unsuccessful initially, then a 0.035-inch guidewire was passed through the endoscope to the duodenum. The forward-viewing endoscope was withdrawn, leaving the guidewire in place. Then, a duodenoscope was advanced over the guidewire with fluoroscopy to reach the papilla, and selective cannulation was achieved successfully.

Additionally, this technique can also be used with balloon enteroscope. We had 2 patients with subtotal or total gastrectomy with Roux-en-Y anastomosis in whom the cannulation of a double-balloon enteroscope was unsuccessful initially, a long-type colonoscope was used at the second attempt by endoscope exchange technique, and selective cannulation was achieved finally.

Itoi et al^[30]^ reported that the endoscope exchange technique was performed with single-balloon enteroscope involving modifying the overtube. In their study, after reaching the papilla, the enteroscope was removed while leaving the overtube in place. A shorter-length gastroscope was inserted to the papilla or bilioenteric anastomosis by a slot created in the side of the overtube at a distance that allows the instrument to extend past the tip of the overtube. As a result, the clinical success was 77% (10/13) for patients with surgically altered anatomy.^[30]^

The endoscope exchange technique has achieved good results; however, further progress and prospective study are expected in the field before becoming a routinely performed procedure.

Previous studies demonstrated that the complication rate ranged from 0% to 19.5% of ERCP in patients with surgically altered anatomy, and perforation was the most frequent and sometimes lethal.^[28]^ Perforations may occur at different levels during the endoscope insertion, leading to abdominal, retroperitoneal, or subcutaneous free air. Difficult endoscope insertion across sharply angulated anastomoses or postoperatively fixed and torqued intestinal limbs may cause perforation along the intestinal tract. When reaching the papilla, perforation may occur during sphincterotomy because of a less well-controlled cutting procedure and an unstable position with a forward-viewing endoscope.

Fortunately, there were no perforations in our study. The complication rate of ERCP in our study was 10.3%, including 3 pancreatitis (mild to moderate), 4 hyperamylasemia, 1 cholangitis, 1 bleeding, and 1 cardiopulmonary accident. All the patients were managed with conventional therapy.

Some limitations of this study are noteworthy, such as its retrospective nature, lack of a control group, and the inclusion of a single-center experience. However, our results demonstrated that ERCP in patients with surgically altered anatomy was safe and feasible. Since there is currently no gold standard approach to deal with biliopancreatic disorders in patients with surgically altered anatomy, further studies comparing the different methods for access to the biliary and pancreatic systems are necessary to guide clinicians in choosing the most effective, safe, and least costly approach.
